# Potential Role of Seaweed Polyphenols in Cardiovascular-Associated Disorders

**DOI:** 10.3390/md16080250

**Published:** 2018-07-28

**Authors:** Manuel Gómez-Guzmán, Alba Rodríguez-Nogales, Francesca Algieri, Julio Gálvez

**Affiliations:** 1 Department of Pharmacology, School of Pharmacy, University of Granada, 18071 Granada, Spain; mgguzman@ugr.es; 2 Instituto de Investigación Biosanitaria de Granada (Ibs.GRANADA), 18071 Granada, Spain; albarnogales@gmail.com (A.R.-N.); fra.algieri@hotmail.it (F.A.); 3 CIBER-EHD, Department of Pharmacology, Centre for Biomedical Research (CIBM), University of Granada, 18071 Granada, Spain

**Keywords:** cardiovascular diseases, hypertension, immune response, metabolic syndrome, seaweed polyphenols

## Abstract

The beneficial effects of various polyphenols with plant origins on different cardiovascular-associated disorders, such as hypertension, diabetes mellitus type 2 and metabolic syndrome are well known. Recently, marine crude-drugs are emerging as potential treatments in many noncommunicable conditions, including those involving the cardiovascular system. Among the active compounds responsible for these activities, seaweed polyphenols seem to play a key role. The aim of the present review is to summarise the current knowledge about the beneficial effects reported for edible seaweed polyphenols in the amelioration of these prevalent conditions, focusing on both preclinical and clinical studies. This review will help to establish the basis for future studies in this promising field.

## 1. Introduction

Cardiovascular diseases (CVDs) constitute the leading cause of disease with a high mortality rate, accounting for one third of global deaths [1]. These comprise different disorders associated with blood vessels and the heart, including coronary artery disease, pulmonary arterial hypertension, deep vein thrombosis and cerebrovascular disease, among others. Several risk factors have been identified as major determinants of these diseases, such as family history, smoking, obesity, dyslipidemia, diabetes mellitus, and hypertension, with the latter being the most prevalent trigger for CVDs [2]. In consequence, they involve large financial consequences at the macroeconomic level, including healthcare spending and national income [3]. Therefore, all efforts to reduce the prevalence of the risk factors for CVDs, using all the available intervention strategies, are very important. Foods are sources of promising bioactive substances that have a potential impact on health. Different studies are starting to reveal the potential use of seaweed polyphenols in cardiovascular conditions. The aim of the present review is to provide scientific arguments supporting the use of seaweed polyphenols for the prevention and potential treatment of CVDs. Different studies, mainly conducted in experimental models and in vitro showing the efficacy of these compounds were the basis of the present review. Moreover, we will analyse the mechanisms that may be involved in the beneficial effects of seaweed polyphenols on CVDs.

## 2. Seaweed Polyphenols

Seaweeds, also known as macroalgae, are an extensive group of macroscopic organisms that comprise a few thousand species of the marine ecosystem. Seaweeds are divided into three main phyla according to their colour: Chlorophyta (green algae), Rhodophyta (red algae) and Phaeophyta (brown algae). The colours of the marine macroalgae are associated with pigments such as chlorophyll for green, phycobilins for red, and fucoxanthin for brown algae [4]. Seaweeds have been consumed as sea vegetables in Asian countries for some thousands of years and are traditionally distinguished for their health benefits. Lately, knowledge of the influence of dietary seaweeds on health and well-being has gain attention due to their unique composition. Several studies have revealed that seaweeds are not only a good source of carbohydrates, dietary fibre, proteins and peptides, vitamins, oils, fats, polyunsaturated fatty acid, and minerals, but also contain a large concentration of antioxidants compounds such as polyphenols [5,6].

**Figure 1:**
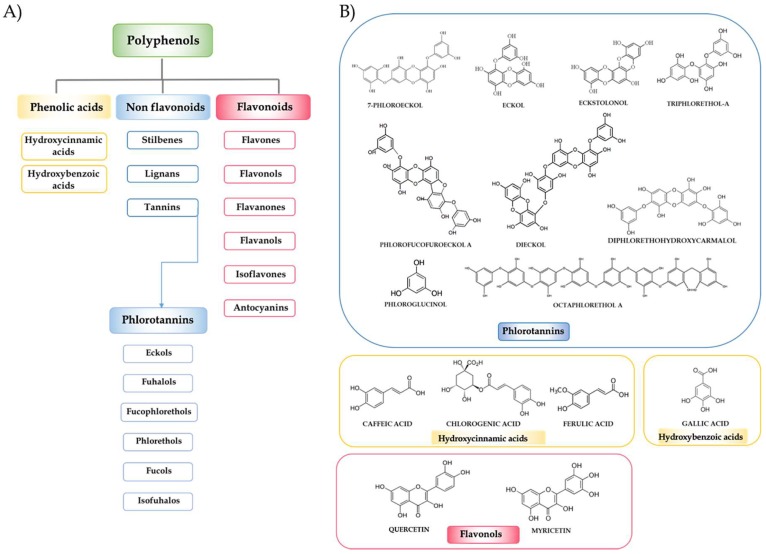
(**A**) Classification of polyphenols and the six major subclasses of tannins in algae, the phlorotannins. (**B**) Chemical structure of some of the main seaweed polyphenols with beneficial effects on cardiovascular diseases.

Polyphenols are a heterogeneous group of compounds, and the major group of phytochemicals found in the human diet, including in fruits, vegetables, seeds, essential oils and their derived foods and beverages. These plant secondary metabolites contain innumerable phenolic structures and differ structurally from simple molecules to highly polymerized compounds. The polyphenol family can be classified by their source of origin, biological function, and chemical structure. To simplify, and according to the chemical structure of the aglycones, polyphenols can be categorized into phenolic acids, flavonoids, stilbenes, lignans and others phenolic compounds. Among them, flavonoids are the most widely distributed and can be divided into six major subclasses: flavonols, flavanols, flavanones, flavones, isoflavones, and anthocyanins (Figure 1) [7]. Epidemiological, clinical and nutritional studies strongly support the evidence that dietary polyphenols play important roles in human health. Their regular consumption has been associated with a reduced risk of different chronic diseases, including cancer, metabolic and neurodegenerative disorders and CVDs.

Marine macroalgae are a rich source of polyphenolic compounds such as catechins, flavonols, and phlorotannins in particular. The largest proportion of phenolic compounds contained in green and red algae are bromophenols, phenolic acids, and flavonoids. On the other hand, phlorotannins, a group of complex polymers of phloroglucinol (1,3,5-trihydroxybenzene), are the dominant polyphenolic secondary metabolites found only in marine brown algae [5,8,9,10,11,12]. Phlorotannins can be classified into six major subclasses: eckols, fuhalols, fucophlorethols, phlorethols, fucols, and ishofuhalols (Figure 1). These compounds are known to exist in soluble or cell-wall-bound forms, and are necessary for the physiological integrity of algae, being also involved in a number of important secondary roles. Brown seaweeds produce these compounds to protect themselves against herbivores and stress conditions, minimising the oxidative damage caused by nutrient deprivation and ultraviolet radiation, among others [13,14,15]. These phytochemicals have attracted much attention because, similar to other polyphenolic compounds, they are bioactive compounds with potential health benefits in numerous human diseases due to their enzyme inhibitory effect and antimicrobial, antiviral, anticancer, antidiabetic, antiallergic and anti-inflammatory activities; however, most of the studies have been focused on their antioxidant activity [14,16].

## 3. Oxidative Stress

Oxidative stress is the excessive concentration of reactive oxygen species (ROS), especially superoxide anion (O_2_^−^). It is a deleterious process that causes cell damage and represents a key feature for the progression of atherosclerosis. Algae have been studied as a source of antioxidants, as evidenced both in vitro and in human subjects [17,18,19]. Athiperumalsami et al. (2010) [20] investigated the antioxidant activity of six species of seaweeds collected from the Gulf of Mannar and they concluded that the seaweeds *Acanthophora spicifera*, *Padina tetrastromatica* and *Ulva lactuca* are effective antioxidants, showing lower half-maximal inhibitory concentration (IC50) values than the standard tocopherol. They also investigated the antioxidant activity of a methanolic extract of *Gracilaria foliifera*, which showed higher efficacy than tocopherol when tested using the H_2_O_2_ scavenging method. Similar results were described by Sivagnanam et al. (2015) [11], who analysed the content in *Saccharina japonica* and *Sargassum horneri* oils.

**Figure 2:**
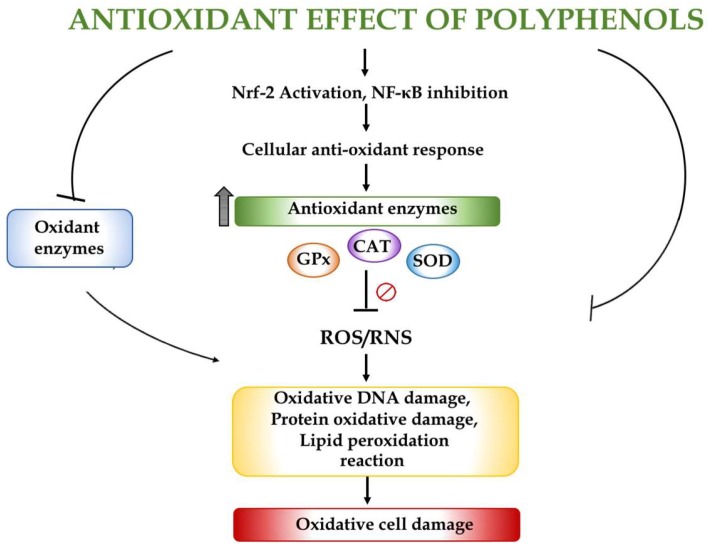
Potential mechanisms contributing to the beneficial effects of polyphenols by protecting cells against oxidative stress. Abbreviations: CAT, catalase; GPx, glutathione peroxidase; NF-κB, nuclear factor kappa-light-chain-enhancer of activated B cells; Nrf-2, nuclear factor erythroid 2-related factor 2; RNS, reactive nitrogen species; ROS, reactive oxygen species; SOD, superoxide dismutase.

The antioxidant properties of seaweed phenolic compounds have been previously reported [21,22]; however, the evaluation of their effects raises several problems when moving from in vitro to the complexity of in vivo systems, due to their different bioavailability and the difficulties in unravelling the intricate mechanisms of absorption and metabolism. Namvar et al. (2013) [23] showed the beneficial effects of a polyphenol-rich seaweed (*Sargassum muticum*) obtained from Persian Gulf waters due to its antioxidant, antiproliferative and antiangiogenic properties. This seaweed has even been proposed as a source of potential complementary and functional food for the prevention of cancer. Antioxidant assays revealed that some algae species from the Danish coast possess a considerable antioxidant activity. These results correlated well, but not only, with the total phenolic content, indicating that seaweed polyphenols are active components in these extracts. *Polysiphonia fucoides* and all the *Fucus* species tested showed the highest radical scavenging activity [24]. More recently, different phenolic antioxidant compounds have been identified by high-performance liquid chromatography (HPLC) in an extract obtained from the brown alga *Himanthalia elongate*, including four phenolic acids (gallic acid, chlorogenic acid, caffeic acid and ferulic acid), two flavonoids (quercetin and myricetin) and phloroglucinol, the most characteristic polyphenol in seaweeds [25]. Similarly, Fernandez Segovia et al. (2018) [26] have determined the total phenolic compound content in four seaweeds, nori (*Phorphyra* sp.), kombu (*Laminaria* sp.), wakame (*Undaria* sp.) and sea spaghetti (*Himanthalia elongata*), and their in vitro antioxidant capacity. In this study, the total phenolic compound content obtained in sea spaghetti was significantly higher than in the rest of the species and this seaweed had the highest value of antioxidant activity measured by the ferric reducing antioxidant power (FRAP) and the 2,2′-azino-bis(3-ethylbenzothiazoline-6-sulfonic acid) (ABTS) methods. In 2016, Fernando et al. [27] reviewed the antioxidant properties of numerous polyphenolic compounds derived from marine algae and described their structure–activity relationship. The antioxidant effect of polyphenols is related to their capacity to enhance the enzymatic activity of different enzymes, including catalase, glutathione peroxidase and superoxide dismutase, their potent free radical scavenging properties and their ability to interact with other molecular targets, as they are capable of reducing arginase-2, and activating the Nrf2/ARE pathway and SIRT-1 [28] (Figure 2).

Among seaweed polyphenols, phlorotannins have also been shown to possess high in vitro antioxidant activity [29,30]. Phlorotannins isolated from the brown alga *Ecklonia cava*, one of the most abundant sources of polyphenolic compounds, revealed antioxidant properties in vitro (Li et al., 2009). Shibata et al. (2007) and Audibert et al. (2010) [31,32] studied the antioxidant activity of semi-purified extracts of phlorotannin fractions and found that low molecular weight phlorotannins exhibited higher antioxidant activity, and this capacity decreased with polymerisation. Most of the studies have been conducted with extracts from algae, whereas the antioxidant capacity of individual phlorotannins has seldom been analysed. A recent study has described the structure-dependant antioxidant capacity of phlorotannins from the brown algae *Fucus vesiculosus* [33]. The authors concluded that the presence of free hydroxyl groups determines the antioxidant capacity of the phlorotannins.

Briefly, polyphenols isolated from marine algae could be a potential source of antioxidants with protective and beneficial effects. Indeed, for a long time, their bioactivity has simply been attributed to their direct antioxidant, radical scavenging and anti-inflammatory properties. However, nowadays, many other mechanisms are considered, including the modulation of intracellular signalling cascades and gene expression, and interaction with gut microbiota [7,28].

## 4. Hypertension

**Figure 3:**
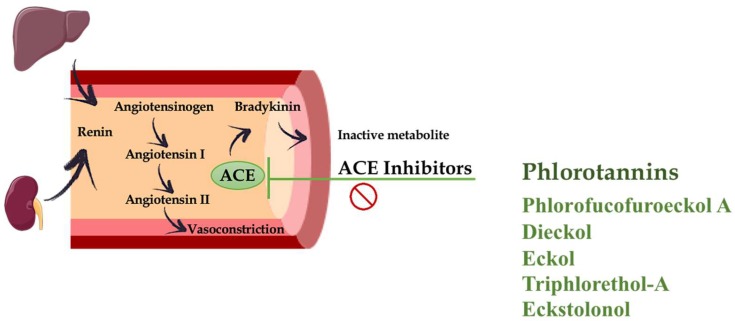
Schematic representation summarizing the potential mechanisms contributing to vasoconstriction reduction subsequent to an inhibition of Angiotensin-I converting enzyme (ACE) in response to several phlorotannins.

High blood pressure (BP) or hypertension is one of the most relevant independent risk factors for CVDs. Hata et al. (2001) [34] reported the positive impact of the consumption of wakame (*Undaria pinnatifida*) on hypertensive patients. Wakame significantly reduced both systolic and diastolic BP. The antihypertensive effect exerted by this alga was ascribed to its content in alginates, a polysaccharide that has shown preventive effects in experimental models of hypertension in rats, in addition to their beneficial impact on cholesterol and diabetes mellitus [35,36]. Several studies have described that bioactive peptides and protein hydrolysates from seaweeds have antihypertensive properties [6,37,38], such as those from *Undaria pinnatifida* [39,40,41], *Palmaria palmata* [42,43,44], *Porphyra columbina* [45,46], *Porphyra yezoensis* [47,48], among others. However, other bioactive compounds found in seaweeds can also contribute to the antihypertensive effect reported for these algae. This could be the case of polyphenols. As mentioned previously, the ability of these polyphenolic compounds to exert antioxidant effects can also account for a suggested antihypertensive activity, similar to that previously described for other phenolic derivatives from land plants, such as flavonoids [49,50]. On the other hand, polyphenols have been described to act as Angiotensin-I converting enzyme (ACE) inhibitors [37,51,52,53]. ACE is a zinc-containing metalloproteinase that catalyses the conversion of angiotensin I to angiotensin II, the latter is a potent vasoconstrictor involved in the pathogenesis of hypertension. ACE also facilitates the degradation of the vasodilator bradykinin. This enzyme has a crucial role in the control of BP (Figure 3). In consequence, its inhibition has become a major target for hypertension control. This explains the development of different ACE inhibitors, which are typically considered one of the main therapeutic strategies for CVDs in humans [54]. When considering the mechanisms involved in this inhibitory effect found in natural compounds, several polyphenolic compounds from plant extracts have been reported to inhibit ACE activity through the sequestration of the enzyme metal factor, Zn^2+^ ion [55,56,57]. Similarly, phlorotannins might be found to be associated with proteins or glycoproteins, forming a complex. This complex inhibits the ACE activity, following a noncompetitive profile. In contrast, commercial ACE inhibitors, such as captopril, show competitive inhibition [53]. In fact, several studies have confirmed this activity for different phlorotannins, such as those present in extracts from *Ecklonia cava* or *Ecklonia stolonifera*, including phlorofucofuroeckol A, dieckol and eckol. These phlorotannins showed similar IC50 values to ACE inhibitors, although these values were much higher (approximately 100-fold) than those found for captopril. Nevertheless, other phlorotannins also found in these algae, such as triphlorethol-A and eckstolonol, have been reported to display lower inhibitory activity [53,58]. In addition to *E. cava* and *E. stolonifera*, other marine algae containing phlorotannins, such as *Lomentaria catenata*, *Lithophyllum okamurae, Ahnfeltiopsis flabelliformis* and *Fucus spiralis* also exert potent ACE inhibitory activity, with similar IC50 values [37,59]. However, despite there being several studies suggesting a positive impact of phlorotannins on hypertension based on their ability to act as ACE inhibitors or due to their antioxidant properties, limited in vivo experiments using rodent models of hypertension are available to confirm this impact.

**Figure 4:**
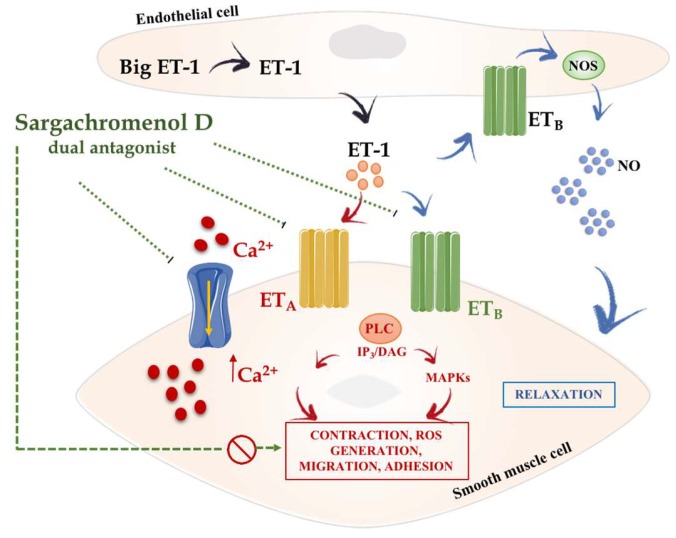
The possible role of sargachromenol D in the prevention of hypertension is most likely due to its ability to act as an antagonist of the endothelin ET_A/B2_ receptors and its capacity to block L-type Ca^2+^ channels. Abbreviations: Ca^2+^, calcium; DAG, diacylglycerol; eNOS, endothelial nitric oxide synthases; ET-1, endothelin-1; ET_A_, endothelin A receptor; ET_B_, endothelin B receptor; IP3, inositol 1,4,5-triphosphate; MAPKs, mitogen-activated protein kinases; NO, nitric oxide; PLC, phospholipase C.

In addition to phlorotannins, farnesylacetones—other constituents from seaweeds—have also been described for their potential use in hypertension due to their reported vasodilator effects. Thus, four compounds isolated from *Sargassum siliquastrum*, including two farnesylacetones, a reduced form of one of these and sargachromenol D, displayed vasodilatory effects on the basilar artery of rabbits [60,61]. These effects were probably related with their ability to effectively antagonize the L-type Ca^2+^ channel [61,62]. In this sense, although several factors are assumed to contribute to hypertension, it is clearly established that an increased vascular tone is always the final target, as this mainly mediated by increased Ca^2+^ influx via L-type Ca^2+^ channels in vascular smooth muscles [63]. In fact, L-type Ca^2+^ channel blockers have been extensively used as antihypertensive agents [54]. In addition to its capacity to block the L-type Ca^2+^ channel, sargachromenol D is also able to antagonise the vasoconstriction induced by endothelin-1 (ET-1), most likely due to its ability to act as an antagonist of the endothelin A and the endothelin B2 (ET_A/B2_) receptors, which can also account for its beneficial effects in hypertension [61] (Figure 4). In fact, ET-1 is a mediator with vasoconstrictor properties that has been reported to play a key role in pathological hypertension, being associated with an increased risk of major cardiovascular and cerebrovascular events [64]. For this reason, selective endothelin receptor type-A antagonists were initially developed for hypertension treatment, although the development of intolerable adverse events limited its use in human therapy, and it is mainly restricted for pulmonary arterial hypertension [65,66]. Given the pharmacological properties of sargachromenol D as a dual antagonist L-type Ca^2+^ channel and endothelin ET_A/B2_ receptor, a dose of 80 mg/kg of sargachromenol D was assayed orally in spontaneous hypertensive rats (SHRs). Sargachromenol D showed acute effects in SHRs by significantly decreasing both systolic and diastolic BP for 24 h after administration [61]. These authors suggested that sargachromenol D could be an appropriate drug for BP control in severe hypertension that is not controlled by conventional combinatorial therapy.

Regarding human studies, there is a large body of evidence supporting the antihypertensive effects of products from land plants containing polyphenols [28]. However, only a few epidemiological studies have examined whether seaweed consumption is associated with a beneficial impact on BP levels, without clearly attributing this effect to the presence of specific components. In fact, their preventive antihypertensive effects have been associated in general with their content in minerals, dietary fibres, peptides or polyphenols [6]. One of the first studies was published in 1991, which described the effects of a potassium-loaded, ion-exchanging, sodium-adsorbing, potassium-releasing seaweed preparation containing dietary fibre in the treatment of mild hypertension. A significant decrease in the mean BP after four weeks of treatment compared to placebo was found in this study [67]. Later, Hata et al. (2001) [34] performed a randomly assigned, case-control study and observed a significant reduction in both systolic and diastolic BP in hypertensive elderly Japanese patients administered with dried *Undaria pinnatifida* powder. A daily dose of 5 g of this powder packed in capsules was administered to these patients for eight weeks. Similarly, a cross-sectional study suggested that seaweed consumption might have beneficial effects on BP measured in 223 boys and 194 girls [68]. Of note, no reduction in BP has been found for normotensive healthy adults when *Undaria pinnatifida* [34] or a polyphenol-rich extract of the brown seaweed *Fucus vesiculosus* [69] were administered.

Hypertension remains a major public health problem although there a large number of drugs are available for its treatment. However, its control has had limited success, and doctors and patients have a growing interest in the use of the dietary approaches to stop hypertension (DASH) diet and natural complementary therapies to reduce the number and/or dose of antihypertensive drugs to manage this condition. As we have described, the polyphenols found in seaweeds, particularly sargachromenol D and phlorotannins, have been proposed to have an important potential in the prevention of hypertension, maybe due to their potent ACE inhibitory activity, antagonism of L-type Ca^2+^ channels or endothelin ET_A/B2_ receptors, as well as to their antioxidant effects. Since many others physiological systems influence BP, it could be interesting to explore whether these polyphenols may act as K^+^ channel openers, Angiotensin II receptor type 2 (AT2) agonists, angiotensin II receptor type 1 (AT1) antagonists, chymase inhibitors, inhibitors of nicotinamide adenine dinucleotide phosphate (NADPH) oxidases, vasopeptidase inhibitors, Rho-kinase inhibitors or NO-releasing agents, all of which are involved in BP control. As mentioned before, although the polyphenol content could be responsible for the antihypertensive benefits of the seaweeds, other components such as peptides, soluble dietary fibres, lipids and minerals contribute. Consequently, it would be interesting to elucidate the specific BP-lowering capacity of the isolated polyphenols in humans.

## 5. Obesity and Diabetes

Obesity and diabetes are considered two of the most prevalent metabolic diseases and they constitute a growing global public health problem, affecting more than 300 million people worldwide. Obesity and diabetes are expected to drastically increase over the next decades [70]. Obesity is characterised by excessive growth and development of adipose tissue when an imbalance between energy intake and energy expenditure takes place [71] and when combined with diabetes, manifested by insulin resistance and impaired fasting glucose and/or impaired glucose tolerance, is also associated with hyperglycemia [72]. A sustained hyperglycemic status can result over time in endothelial dysfunction and the subsequent development of a vascular inflammatory response, thus promoting accelerated atherosclerosis and the onset of cardiovascular events [73].

Different epidemiological studies and dietary interventions have suggested that high phenolic intake from edible sources, including cocoa, grape, tea or extra virgin olive oil, can be linked to a decreased risk of human CVDs [74,75,76,77]. However, most of the beneficial effects of seaweeds reported in obesity and diabetes are based on preclinical studies. Thus, the commercially available microalgae from the genus *Chlorella* or genus *Spirulina* have been described to exert a beneficial impact on different experimental models of obesity in rodents [78,79]. In fact, double-blind crossover studies have shown that the intake of *Spirulina* (about 2.8 g per day) for approximately one month reduces blood-sugar levels in obese outpatients [80]. Similarly, AbouZid et al. (2014) [81] reported the screening of four crude extracts of Egyptian algae traditionally used for the improvement of diabetes mellitus (*Caulerpa lentillifera*, *Enteromorpha intestinalis*, *Spirulina versicolor* and *Ulva lactuca*) in an experimental model of type 2 diabetes in mice, revealing that all of them had antihyperglycemic effects. However, in these studies, the observed beneficial effects were not ascribed to the presence of specific components in the algae. Moreover, the ability of *Spirulina* to exert in vivo antihyperglycemic activity in experimental models of diabetes in rats has been described, most likely involving the antioxidant properties ascribed to some of their components, such as C-phycocyanins or β-carotene [82,83].

In order to determine the correlation between the phenolic content of algae and their potential application in controlling diabetes and obesity, different phenolic-rich extracts from edible marine algae have been assayed in vitro by evaluating their impact on the activity of specific enzyme assays (α-amylase or α-glycosidase). In fact, different drugs able to inhibit these intestinal enzyme activities, such as acarbose or miglitol, are currently used in human therapy for their antidiabetic activity. Thus, the phenolic extracts from *Alaria esculenta*, *Ascophyllum nodosum*, *Palmaria palmate* and *Sargassum hemiphyllum* showed similar antioxidant abilities, and were able to inhibit α-amylase activity [84,85]. Of note, the extract from *Ascophyllum nodosum* showed the lowest IC50 value, even lower than that obtained for acarbose, an inhibitor of α-glucosidase activity, which is currently used for this purpose in human therapy [84]. Inhibition of α-amylase has also been reported for the crude phlorotannin-rich fraction extracted from the brown alga *Ecklonia cava*, and was considerably more effective than the most potent single component, the phlorotannin dieckol. This suggests synergetic interactions between components regarding their biological activities [86,87]. Similarly, phlorotannins and bromophenols have been considered the active components responsible for the in vitro inhibition of α-glucosidase activity exerted by extracts from several brown algae (*Ascophyllum nodosum*, *Ecklonia* sp., *Eisenia* sp., *Fucus vesiculosus*, *Ishige okamurae*, *Ishige foliacea*, *Laminaria digitata*, *Laminaria japonica*, *Leathesia nana*, *Sargassum patens*), red algae (*Rhodomela larix, Rhodomela confervoides)* and green algae (*Avrainvillea longicaulis*) [14,88,89,90]. Moreover, it has been reported that the ability of naturally occurring polyphenols and phlorotannins from some species of brown seaweeds (*Ascophyllum nodosum*, *Fucus vesiculosus, Ishige foliacea*) to inhibit α-amylase and α-glucosidase enzymes in vitro is correlated with a reduction in carbohydrate absorption in vivo. These findings support the potential use of these compounds against type 2 diabetes [90,91].

Other enzyme activity that has prompted interest as a target in the management of diabetes is protein-tyrosine phosphatase 1B (PTP1B). This enzyme acts as a negative regulator of insulin signalling and it is located in the endoplasmic reticulum membrane of hepatic, muscular and adipose tissues [92]. Different phlorotannins, including eckol, phlorofucofuroeckol-A, dieckol and 7-phloroeckol as well as bromophenols have been described as potently inhibiting this enzyme activity in a noncompetitive way [93,94]. This inhibition supports their potential benefits in diabetes associated with their ability to improve insulin sensitivity.

Moreover, additional in vitro assays have been performed with phenolic-enriched extracts from seaweeds on adipocytes, such as the 3T3-L1 cell line, in order to evaluate the impact of these extracts on cell activity and inflammatory status. Accordingly, the ethyl-acetate extract from *Fucus distichus* containing phloratannin oligomers has been reported to inhibit lipid accumulation by promoting lipolysis in these cells. Furthermore, in these adipocytes, this extract dose-dependently reduced the gene expression levels of the pro-inflammatory cytokine TNF-α (tumour necrosis factor alpha), the chemokine MCP-1 (monocyte chemoattractant protein-1), the adhesion molecule ICAM (intercellular adhesion molecule 1) and the adipokine leptin, whereas it increased the expression of the anti-inflammatory adipokine adiponectin [95]. These changes would suggest that this phenolic-enriched extract could ameliorate the inflammatory status reported to occur in adipose tissue, which clearly contributes to the development of cardiovascular events in obese patients. In addition, an aqueous ethanolic extract of *Ascophyllum nodosum* was found to stimulate basal glucose uptake into 3T3-L1 adipocytes. This effect was associated with the presence of polyphenolic components in the extract [96], which can be complementary to the effects observed with this alga in reducing the carbohydrate absorption in vivo by inhibiting α-amylase and α-glucosidase enzyme activities [91].

**Figure 5:**
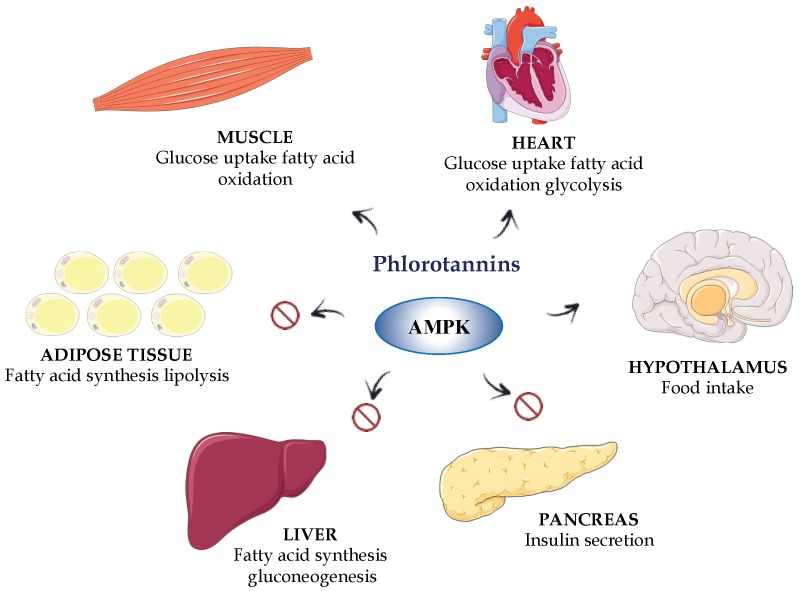
An integrated overview of the beneficial effects of phlorotannins through the activation of AMP-activated protein kinase (AMPK) and the regulation of its downstream genes. Abbreviation: AMPK, 5′-adenosine monophosphate-activated protein kinase.

Different in vivo studies in experimental models of metabolic syndrome in rodents have confirmed a beneficial effect of phenolic-containing extracts from seaweeds on obesity and diabetes. The polyphenol-enriched extract from *Ecklonia cava* is one of the most extensively studied in these preclinical models. This extract has been shown to ameliorate insulin resistance, glucose tolerance and lipid metabolism in a high-fat diet (HFD)-induced obesity model in mice. Different mechanisms seem to be involved in these beneficial effects, involving the down-regulation of the obesity-associated inflammatory response due to the well-reported antioxidant properties, which result in the activation of AMP-activated protein kinase (AMPK) and the regulation of its downstream genes [97,98,99] (Figure 5). The AMPK is considered a key metabolic modulator; the activity of AMPK is compromised in diabetes, as this activity is regulated by the drugs currently used in human therapy as antidiabetics, such as metformin [100]. Moreover, this polyphenolic-enriched extract has also been shown to decrease the expression of hepatic lipogenesis-related genes that were upregulated in HFD mice, thus improving the altered lipid metabolism in obese animals [101]. Similarly, the oral administration of an extract from the brown alga *Ishige okamurae* to db/db mice, a model of type 2 diabetes, significantly suppressed the increase in the fasting blood glucose concentration and ameliorated the altered insulin sensitivity in comparison with the untreated control group. The effects found were similar to those found for rosiglitazone, a peroxisome proliferator-activated receptor-γ (PPARγ) agonist currently used in the treatment of human type 2 diabetes [102]. In fact, the effects exerted by this extract were associated with an improvement in the activity of the different enzymes involved in glucose metabolism, including glucokinase activity, glucose-6-phosphatase and phosphoenolpyruvate carboxykinase, thus supporting its beneficial impact on insulin resistance in diabetic mice [102].

The effects observed in these in vivo assays were ascribed to the presence of specific bioactive compounds, such as phlorotannins, which include phloroglucinol, eckol, dieckol and their derivatives [103]. The phlorotannins 2,7″-phloroglucinol-6,6′-bieckol, phlorofucofuroeckol A and diphlorethohydroxycarmalol have been reported to reduce postprandial hyperglycaemia in diabetic mice, an effect most probably related to their ability to inhibit α-amylase or α-glycosidase [104,105,106], as mentioned above for these and other algae constituents. Similarly, the oral administration of dieckol was able to reduce postprandial hyperglycaemia in streptozotocin-induced diabetic mice [107]. Moreover, when this phloratannin was administered intraperitoneally in the db/db mouse model of type 2 diabetes, significantly lower blood glucose and insulin concentrations were found compared to the untreated control group [108].

However, in addition to the inhibition of the intestinal glucose absorption, these phlorotannins can exert direct effects on different target tissues in obesity and diabetes, liver, skeletal muscle and adipose tissue, which can also account for their beneficial profile in these conditions. Therefore, phloroglucinol has been reported to decrease glucose production in mouse primary hepatocytes. In addition, this compound has been shown to reduce the hepatic expression of phosphoenol pyruvate carboxykinase and glucose-6-phosphatase, both enzymes involved in gluconeogenesis, or to increase AMPK activity in HepG2, a hepatocyte cell line [109]. Similarly, it has been reported that 2,7″-phloroglucinol-6,6′-bieckol was able to protect INS-1 cells, a pancreatic beta cell line, against high glucose-induced apoptosis [87]. As a result, this compound could preserve the beta pancreatic cell function from the toxic effect that hyperglycaemia can exert on these cells in type 2 diabetes, which has been implicated in the pathogenesis of this metabolic condition [110]. In addition, in the alloxan-induced hyperglycaemia zebrafish model, dieckol showed an antidiabetic effect. This effect was evidenced by a significant reduction in blood glucose concentrations, which was associated with an improvement of the hepatic glucose metabolism. An improvement of the glucose uptake in muscle tissues was also found, this occurred through the stimulation of the muscle PI3k-Akt pathways, which are downregulated in situations of insulin resistance [111]. Similarly, dieckol has been shown to exert beneficial effects on the adipose tissue in experimental diabetes, since it was able to reduce the lipid accumulation that occurs in the adipocytes in zebrafish and mice fed a high-fat diet [112]. This effect was associated with the activation of AMPK signalling, which resulted in the inhibition of lipid synthesis, as observed in 3T3-L1 cells and in the mouse model of obesity.

Moreover, dieckol reduced the expression of early adipogenic genes in 3T3-L1 cells, which resulted in the down-regulation of late adipogenic factors and a decrease in triacylglycerol content in these cells. In addition, dieckol inhibited the mitotic clonal expansion of adipocytes, via cell-cycle arrest [112]. Furthermore, the phlorotannin octaphlorethol A, isolated from *Ishige foliacea*, was reported to exert beneficial effects on hyperglycaemia by improving hyperinsulinemia and to impair glucose tolerance in db/db mice. This response was associated with an increase in GLUT4-mediated glucose utilization via the activation of AMPK in muscles and the suppression of hepatic gluconeogenesis by inhibiting the enzymes glucose-6-phosphatase and phosphoenolpyruvate carboxykinase activity, which are upregulated in diabetes [113].

**Figure 6:**
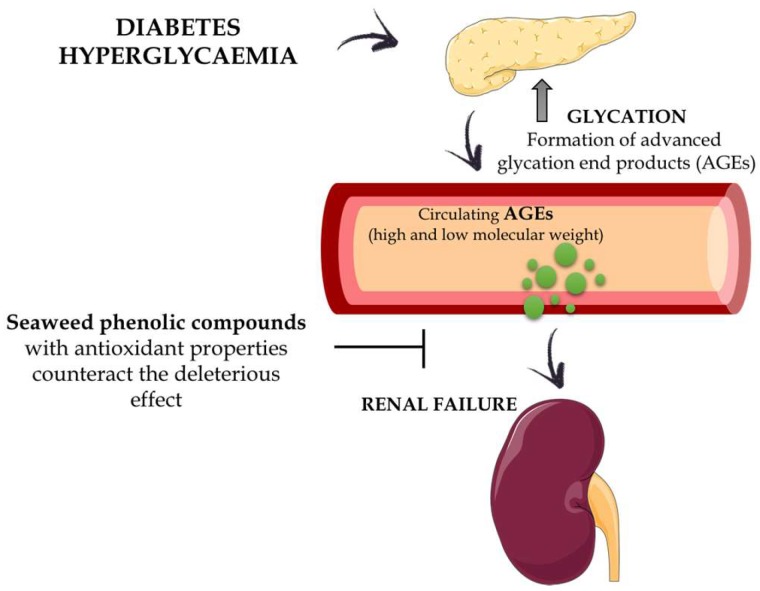
Schematic representation showing the antioxidant properties of seaweed phenolic compounds against the toxic effects associated with the hyperglycaemia occurring in diabetes mediated by the formation of advanced glycation end products (AGEs).

Finally, it is well documented that the toxic effects associated with the hyperglycaemia occurring in diabetes can be mediated by the formation of advanced glycation end products (AGEs). The AGEs, either directly or through the generation of other reactive intermediates, are responsible for the damage in different tissues and organs in these patients, including diabetic nephropathy [114]. The antioxidant properties of seaweed phenolic compounds previously described could counteract the deleterious effects of the AGEs (Figure 6).

This statement is supported by the fact that diphlorethohydroxycarmalol, a polyphenol isolated from the edible seaweed *Ishige okamurae*, was able to protect HEK cells, a human embryonic kidney cell line, against methylglyoxal-induced oxidative stress, a highly reactive carbonyl metabolite of glucose and a major precursor of AGEs [115]. In addition, the association between enhanced hyperglycaemia in diabetes and oxidative stress is well known. Oxidative stress is considered a key mechanism for glucose toxicity in pancreatic β cells [116]. However, it is not clear if the above-mentioned antioxidant properties exhibited by phlorotannins are the only mechanism involved in the prevention of the deleterious effect that high blood glucose concentrations exert on pancreatic β cells. Thus, 6,6′-bieckol, octaphlorethol-A and dieckol, have shown protective effects against high-glucose-induced toxicity in rat insulinoma cells or in streptozotocin-treated pancreatic β cells, which were associated with a significant reduction in thiobarbituric acid reactive substance (TBARS) concentrations, intracellular ROS or NO. In addition, these compounds reduced the expression levels of the pro-apoptotic protein Bax, which is highly expressed under high glucose concentrations, or increased the expression of the anti-apoptotic protein Bcl-2, thus increasing cell viability in this adverse cell environment [99,117,118]. Furthermore, the phlorotannins dieckol and octaphlorethol-A have been described to increase the activity of antioxidant enzymes, such as catalase, superoxide dismutase and glutathione peroxidase, which can also account for its cell protective effects in high-glucose concentrations [99,117].

All these studies can provide substantial support to the reported beneficial impact that dietary algae consumption may have in decreasing the risk of diabetes mellitus, such as a survey performed in Korean men [107]. Unfortunately, currently little information is available about the effects that seaweed polyphenols can exert in human diabetes. To date, only two clinical studies have evaluated the impact of these compounds on postprandial glycaemic responses in humans. One of these studies evaluated sixty three adults with elevated fasting plasma glucose, finding a significant reduction in two-hour postprandial blood glucose concentration in the intervention group, who received a diet supplemented with a dieckol-rich extract from the brown algae *Ecklonia cava* for 12 weeks, compared to a placebo [119]. The other study included twenty-three participants and evaluated the impact of a commercially available blend of brown seaweed (*Ascophyllum nodosum* and *Fucus vesiculosus*), able to inhibit α-amylase and α-glucosidase activities, on glucose and insulin concentrations over a period of 3 h postcarbohydrate ingestion. The results of this study revealed that, in comparison with a placebo, the consumption of seaweed was associated with an improvement in the insulin sensitivity, although no significant effect was observed on the glucose response [120]. In contrast, a recent study that investigated the impact of a single dose of a polyphenol-rich seaweed extract from *Fucus vesiculosus* on postprandial glycaemia in healthy adults did not show any significant lowering effect on postprandial glucose or insulin responses when compared with a placebo [69].

Finally, despite the fact that the supplementation of humans with polyphenol-rich extracts from algae has been shown to exert some positive effects on fasting blood glucose, total cholesterol and LDL-cholesterol, a recently published systematic review concluded that it was not possible to clearly establish whether these are consistent effects [121]. These authors suggested that additional randomised-controlled trials are required to understand the exact role that marine polyphenols may play in reducing the risk factors for chronic diseases, such as metabolic syndrome, in humans and in preventing the deleterious impact that this condition exerts on cardiovascular function.

Briefly, the marine polyphenol compounds mechanisms can be compared to some anti-diabetic drugs due to their ability to inhibit carbohydrate carbolytic enzymes and PTP1B; to attenuate AGE formation, as well as to act against obesity and the associated inflammatory responses. Taken together, they can be employed in disease management as part of the dietary intake or as purified pharmacological agents and supplements. However, further research is required to make the most of them in preventing and managing these pathologies.

## 6. Present and Future Perspectives: Clinical Application

Cardiovascular diseases (CVDs) are the major cause of death worldwide. A great effort has been made during the last decades to control these CVDs. Although the combination of lifestyle modifications and drug therapy has shown efficacy in most cases, there is a continuing need to identify new complementary targets for the treatment and prevention of CVDs, derived from the fact that the adherence to these treatments is often poor [122]. Different factors account for this poor adherence: a large proportion of hypertensive patients do not respond conveniently to current therapeutic medications, the high cost of medications, their undesired side effects, in addition to their low accessibility and availability in many cases, among others. In this context, patients suffering from CVDs often seek alternative approaches, with herbal remedies being one of the most commonly used alternatives, mainly because herbals are considered a cheaper alternative with fewer side effects than marketed antihypertensive drugs [123]. Several plant drugs have been traditionally used against cardiovascular conditions and some of their beneficial health effects are supported by clinical trials, including garlic (*Allium sativum*), tea (*Camellia sinensis*), hawthorn (Crataegus spp.), saffron (*Crocus sativus*), ginseng (*Panax ginseng*) and roselle (*Hibiscus sabdariffa*) [124,125]. Given the chemical complexity reported for these plants, many compounds have been proposed as the active components responsible for these beneficial effects against cardiovascular conditions in humans. Among these compounds, a prominent role has been ascribed to different polyphenols, including flavonoids, which have been suggested to improve the altered function in various target organs in CVDs through different mechanisms [28]. Of note, flavonoids possess antioxidant properties that ameliorate vascular dysfunction via increasing nitric oxide (NO) bioavailability and endothelium-dependent vasorelaxation [126]. Additionally, these polyphenols are able to inhibit the production of different mediators that directly promote vasosconstriction, such as angiotensin II (AngII) or endotelin-1 (ET-1). Likely, this inhibition is caused by the ability of flavonoids to interfere with the enzyme activity involved in the synthesis of these mediators or with the corresponding signalling pathways in vessels [127,128]. Closely related to the biological properties attributed to flavonoids, these polyphenols have been reported to exert anti-inflammatory effects and suppress the proliferation activity of cancer cells, which can also contribute to their antiatherosclerotic and antihypertensive effects [129,130]. In addition, flavonoids have been reported to exert an antiaggregant effects, thus counteracting the platelet hyperactivity that may characterise CVDs and contribute to their beneficial effects [131]. Moreover, flavonoids have been described to possess beneficial metabolic effects in obesity and atherosclerosis, which can also account for their potential use in CVDs [132,133]. However, despite a great scientific effort having been made to validate the use of natural products obtained from plant extracts in these CVDs, there is a clear demand for the discovery of novel compounds able to improve the efficacy and tolerability of the currently known products. This could be the case for seaweeds, which still remain largely unknown.

Marine algae hold great interest for the development of new drugs and healthy foods. A diet rich in marine macroalgae has been associated with longevity and seaweeds are widely regarded as having the potential to prevent or exert beneficial effects on different diseases, including cardiovascular-associated disorders such as hypertension, diabetes mellitus type 2 and obesity [69]. Among their constituents, peptides, soluble dietary fibres, lipids, minerals and polyphenols are the major bioactive compounds with demonstrated health benefits. Despite polyphenols having very low bioavailability due to their reduced absorption and rapid metabolism; several epidemiological studies have found an inverse association between polyphenols and different diseases. Moreover, numerous biological activities have been attributed to algae polyphenols in vitro. Nevertheless, the cardiovascular effects of edible seaweed polyphenols have not been consistently analysed in vivo or in clinical interventional studies. The effects on CVDs may be attributable to their direct antioxidant activity, and to the anti-inflammatory and vasodilatory effects. In particular, phlorotannins, a class of marine-exclusive polyphenols, have been reported to exert antihypertensive effects acting as ACE inhibitors and showing antioxidant properties through scavenging ROS or the enhancement of antioxidant defences. Phlorotannins have also demonstrated antidiabetic properties through their ability to inhibit different enzymes such as α-amylase and α-glucosidase (decreasing carbohydrate absorption) and PTP1B (improving insulin sensitivity). Phlorotannins have shown protective effects on β-pancreatic cells and adipocytes against apoptosis, oxidative stress and anti-inflammatory effects through decreasing different pro-inflammatory mediators (TNF-α, MCP-1, ICAM and leptin), ameliorating AMPK activity and its downstream genes, and improving the altered lipid metabolism. However, most of the available research has been conducted on non-human subjects. At this point, several questions should be addressed. First, whether these protective effects are related to the isolated polyphenols from seaweeds. To determine this, some approaches studying the cardiovascular effects of isolated pholorotannins from edible seaweeds in animal models and human beings are needed, reporting mechanisms and describing the supporting clinical data. Second, clinical pharmacokinetic studies performed with polyphenol seaweeds are needed, since bioavailability greatly differs from one polyphenol to another and among dietary sources depending on the forms they contain. The analysis of active metabolites (with a special interest in conjugated polyphenols) in plasma and target tissues could be interesting. Third, epidemiological studies should be performed to analyze whether certain types of seaweeds can have greater beneficial effects than others. Fourth, it should be determined whether there are differences between the effects of unprocessed seaweeds, seaweeds enriched with certain polyphenols or polyphenol-rich seaweed extracts. Fifth, due to recent findings, it should be reasonable to study whether algae intake modifies the microbiota and its metabolic by-products towards a beneficial profile, because changes of the gut microbiota may lead to cardioprotective effects. Sixth, although the consumption of polyphenol products is supposed to be safe, it is necessary to clearly determine whether adverse events occur during long term or high dose administration. Addressing all these questions together, it can be elucidated whether seaweed polyphenols can be considered an alternative therapeutic strategy, either to complement or to replace existing conventional medicine approaches to cardiovascular disease.

## 7. Conclusions

Increased consumption of polyphenol-rich seaweeds has been associated with a reduced risk of many diseases due to their numerous possible biological properties. However, more research is necessary to elucidate the mechanisms responsible for the beneficial effects of polyphenol-rich seaweeds on CVDs, to investigate their clinical relevance and to elucidate whether macroalgae represent promising candidates for the development of drugs, functional foods or nutraceuticals useful against cardiovascular-associated disorders. In addition, more toxicological studies are needed to determine the safety of these compounds. While additional randomized trials are ongoing to corroborate their beneficial effects, the current evidence suggests that seaweed polyphenols are a good option for the amelioration of cardiovascular-associated disorders.
